# Transarterial chemoembolization of hepatocellular carcinoma in a rat model: the effect of additional injection of survivin siRNA to the treatment protocol

**DOI:** 10.1186/s12885-016-2357-3

**Published:** 2016-05-23

**Authors:** Thomas J. Vogl, Elsie Oppermann, Jun Qian, Ulli Imlau, Andreas Tran, Yousef Hamidavi, Huedayi Korkusuz, Wolf Otto Bechstein, Nour-Eldin Abdel-Rehim Nour-Eldin, Tatjana Gruber-Rouh, Renate Hammerstingl, Nagy Naguib Naeem Naguib

**Affiliations:** Institute for Diagnostic and Interventional Radiology, Johann Wolfgang Goethe-University, Theodor-Stern-Kai 7, Frankfurt, 60590 Germany; Department of General Surgery, Johann Wolfgang Goethe-University, Frankfurt, Germany; Department of Diagnostic and Interventional Radiology, Faculty of Medicine, Cairo University, Cairo, Egypt; Department of Diagnostic and Interventional Radiology, Faculty of Medicine, Alexandria University, Alexandria, Egypt

**Keywords:** Hepatocellular carcinoma, Survivin siRNA, Chemoembolization

## Abstract

**Background:**

Transarterial chemoembolization is one of the most widely accepted interventional treatment options for treatment of hepatocellular carcinoma. Still there is a lack of a standard protocol regarding the injected chemotherapeutics. Survivin is an inhibitor of Apoptosis protein that functions to inhibit apoptosis, promote proliferation, and enhance invasion. Survivin is selectively up-regulated in many human tumors. Small interfering RNA (siRNA) can trigger an RNA interference response in mammalian cells and induce strong inhibition of specific gene expression including Survivin. The aim of the study is to assess the effectiveness of the additional injection of Survivin siRNA to the routine protocol of Transarterial Chemoembolization (TACE) for the treatment of hepatocellular carcinoma in a rat model.

**Methods:**

The study was performed on 20 male ACI rats. On day 0 a solid Morris Hepatoma 3924A was subcapsullary implanted in the liver. On day 12 MRI measurement of the initial tumor volume (V1) was performed. TACE was performed on day 13. The rats were divided into 2 groups; Group (A, *n* = 10) in which 0.1 mg mitomycin, 0.1 ml lipiodol and 5.0 mg degradable starch microspheres were injected in addition 2.5 nmol survivin siRNA were injected. The same agents were injected in Group (B,=10) without Survivin siRNA. MRI was repeated on day 25 to assess the tumor volume (V2). The tumor growth ratio (V2/V1) was calculated. Western blot and immunohistochemical analysis were performed.

**Results:**

For group A the mean tumor growth ratio (V2/V1) was 1.1313 +/− 0.1381, and was 3.1911 +/− 0.1393 in group B. A statistically significant difference between both groups was observed regarding the inhibition of tumor growth (*P* < 0.0001) where Group A showed more inhibition compared to Group B. Similarly immunohistochemical analysis showed significantly lower (*p* < 0.002) VEGF staining in group A compared to group B. Western Blot analysis showed a similar difference in VEGF expression (*P* < 0.0001).

**Conclusion:**

The additional injection of Survivin siRNA to the routine TACE protocol increased the inhibition of the hepatocellular carcinoma growth in a rat animal model compared to regular TACE protocol.

## Background

Being one of the most common malignancies in the world Hepatocellular carcinoma (HCC) is estimated to be responsible for about one million deaths per year. Owing to its rapid infiltrating growth and complicating liver cirrhosis HCC usually have a poor prognosis [1]. Transarterial chemoembolization (TACE) is a widely accepted therapeutic option for HCC [2]. Survivin is an inhibitor of Apoptosis protein that functions to inhibit apoptosis, promote proliferation, and enhance invasion [3]. Under normal conditions Survivin is expressed in embryonic and fetal tissues and is barely detectable in most differentiated normal adult tissues [4]. In addition, it has been shown that Survivin expression is present in some normal adult tissue cells like T-Lymphocytes, polymorphonuclear-neutrophils, primitive hematopoietic cells and vascular endothelial cells [5, 6].

Survivin is selectively up-regulated in many human tumors, an overexpression of Survivin correlates with poor outcome, treatment resistance [3] reduced disease free survival and overall survival of cancer patients [7]. It has been shown to increase resistance of tumor tissue to apoptotic stimuli mainly through a Caspase-dependent mechanism [5]. Small interfering RNA (siRNA) can trigger an RNA interference response in mammalian cells and induce strong inhibition of specific gene expression hence they can be used to inhibit cancer-related genes including Survivin [8].

In-spite of the wide acceptance of TACE a meta-analysis [9] has demonstrated that 4 of the 6 randomized controlled studies included in the analysis comparing TACE with untreated controls failed to show any impact of TACE on patient survival. Keeping into consideration that there is a great controversy regarding the best chemotherapeutic agents for TACE [10] the need for introducing new chemotherapeutic agents targeting HCC is critical.

Thus, the aim of the current study was to assess the therapeutic efficacy of the additional transarterial injection of Survivin siRNA to the regular TACE protocol compared to the regular TACE protocol in an animal model of hepatocellular carcinoma, and its effect on tumor growth, angiogenesis and vascular endothelial growth factor expression (VEGF) levels.

## Methods

### Animals and tumor cells

All of the experiments on animals were approved by the Regional Administrative Authority in Darmstadt, Germany.

Twenty inbred male ACI-rats (Harlan Winkelmann; Borchen, Germany) weighing 220–240 g were used. The animals were kept under conventional conditions with a temperature of 22 ± 2 °C, a relative humidity of 55 ± 10 %, a dark-light rhythm of 12 h, and they were fed with standard laboratory chow and tap water ad libitum. The hepatoma cell line used in the current study was obtained from the German Cancer Research Center (DKFZ; Heidelberg, Germany). The injected cell line (Morris hepatoma 3924A) represents a rapidly growing, poorly differentiated hepatocellular carcinoma.

### Chemotherapeutics and agents

For TACE a dose of 0.1 mg Mitomycin (Roche, Grenzach-Wyhlen, Germany) was dissolved in 0.1 ml 0.9 % NaCl solution 10 min before application. The embolization was performed using a dose of 0.1 ml lipiodol (Guerbet GmbH, Sulzbach, Germany) and 5.0 mg degradable starch Microspheres (Spherex©, Pharmacia, Erlangen, Germany).

Survivin siRNA was provided from Ruibo Gentech Co (Wuhan, PR. China). The target sequence of Survivin siRNA is as follows: CCGAGAATGAGCCTGATTT. A dose of 2.5 nmol Survivin siRNA was stable at 2–8 °C for 10 min before administration.

### Anesthesia

A combination of intra-peritoneal injection of Ketamine Hydrochloride (Ketanest Parke-Davis, Germany; 100 mg/kg), Xylazine Hydrochloride (Rompun, Bayer Germany; 15 mg/kg) and Atropine Sulfate (Atropin Sulfat Braun, Braun, Germany; 0.1 mg/kg) was used for anesthesia in all Orthotopic, interventional and imaging procedures.

### Tumor implantation (day 0)

The technique for tumor implantation was basically similar to that described by Yang et al. [11] with minor modifications [12]. The Morris Hepatoma 3924A tumor tissue, recovered from the passaged animals 12 days after subcutaneous implantation (corresponding to 5 × 10^6^ tumor cells), was cut into small cubes about 2 mm^3^. A small sub-capsular incision on the left lateral lobe of the liver was made in the recipient ACI-rats under anesthesia. The tumor fragment was gently placed into created pocket with a small cotton swab on the liver surface and the abdominal wall was then closed.

### Interventional therapy (day 13)

For interventional studies a second laparotomy was performed. By using a binocular operative microscope (M651, Leica; Wetzler, Germany), a PE-10 polyethylene Micro-catheter (inner diameter 0.28 mm, outer diameter 0.61 mm; Wenzel; Heidelberg, Germany) was retrogradely inserted into the Gastro-duodenal artery and pushed forward to the hepatic artery. Different agents were then injected into the hepatic artery using the sandwich technique (subsequent injection of Mitomycin +/− Survivin siRNA + Lipiodol + degradable starch Microspheres) within 20 min. Each group of animals received treatment according to the following protocols:

- Group A (TACE + Survivin siRNA, test group, *n* = 10): Mitomycin (0.1 mg) + Lipiodol (0.1 ml) + degradable starch Microspheres (5.0 mg) + Survivin siRNA (2.5 nmol)
- Group B (TACE alone, control group, *n* = 10): Mitomycin (0.1 mg) + Lipiodol (0.1 ml) + degradable starch Microspheres (5.0 mg)

### MR imaging and analysis (day 12 and 25)

MRI imaging before (on day 12) and after (on day 25) treatment was performed using a 3.0 Tesla MRI unit (Magnetom, Siemens; Erlangen, Germany) by using a wrist coil (Small field of view). T1-weighted (SE: TR/TE, 500/12 ms) and T2-weighted (TSE: TR/TE, 3870/80 ms) transverse images with a section thickness of 2 mm and 184 × 256 matrix were acquired. There was no gap between sections and no contrast medium was administered. The tumor volume was determined and evaluated in the T2-weighted image according to the formula [13]: V = 0.5 × d1 × d2^2^, where d1 is the maximum diameter of the tumor and d2 is the minimum diameter perpendicular to d1. Image evaluations and size assessments were performed by a single radiologist with more than 15 years’ experience in abdominal MRI imaging and who was blinded to the group assignment of the animal in the study.

### Western blot (day 25)

Western blot analysis was carried out to determine the expression level of the VEGF in the two groups. After the MRI examination, all the rats were sacrificed using an over-dose of intravenous Sodium Pentobarbital. To homogenize the tumors, Precellys Homogenizer IV (Peqlab Biotechnologie GmbH; Erlangen, Germany) at 4 °C was used in a lysis buffer which is composed of 50 mM HEPES, 200 mM NaCl, 0.2 mM MgSO4, 0.4 mM Phenylmethylsulfonyl fluoride, 2 % Triton- X-100, 10 μg/mL Leupeptine, 10 μg/mL Aprotinine, 0.02 % soybean trypsin inhibitor and 0.2 mM Orthovanadate (Sigma-Aldrich; Taufkirchen, Munich, Germany). The resulting Cell lysates were centrifuged for 10 min at 12,000 × g at 4 °C. Coomassie Plus protein assay kit (Pierce; Rockford, IL, USA) was used to measure the protein concentration in the supernatants. The protein concentration results were obtained Spectrophotometrically by Tecan Infinite® M 200 microplate reader (Tecan-Deutschland; Crailsheim, Germany) at 595 nm. Protein was then denatured in Laemmli sample buffer (Bio-Rad Laboratories; Munich, Germany) with β mercaptoethanol (Sigma; Taufkirchen, Germany), boiled for 5 min, and transferred on ice. Sodium Dodecyl Sulfate Polyacrylamide gel electrophoresis (SDS-PAGE) (50 μg per lane) was then conducted. The molecular weight standards used were PeqGold prestained protein markers IV (Peqlab Biotechnologie GmbH; Erlangen, Germany). After separation using gel electrophoresis, protein was blotted onto a Polyvinylidene Difluoride membrane (Hybond P; GE Healthcare; Munich, Germany). Blots were then blocked with 10 % low-fat milk for 1 h at room temperature followed by overnight incubation at 4 °C with primary antibody from Santa Cruz Biotechnology (Rabbit polyclonal VEGF 1:200; Rabbit polyclonal MMP-9 and mouse monoclonal β Actin, (clone AC-15, 1:1000; Sigma). Blots were then washed 3 times with Towbin buffer with 0.5 % Tween 20 followed by incubation for 30 min at room temperature with secondary antibody from Millipore GmbH; Schwalbach/Ts, Germany (Polyclonal goat Anti-rabbit IgG, 1:5000; goat Anti-mouse IgG, 1:5000, both HRP conjugated). All antibodies were diluted in Towbin Buffer with 0.5 % Tween 20 and 0.5 % bovine serum albumin. Blots were then washed and incubated withEnhanced Chemiluminescence detection kit (GE Healthcare; Munich, Germany). Signal intensity was finally detected and captured by Fusion FX-7 (Vilber Lourmat, Marnee la Vallee, France), documented and analyzed by Bio1D software (Vilber Lourmat). β Actin was used as the loading control.

### Immunohistochemical examination (day 26)

The Liver samples were embedded and frozen in a Tissue-Tek (Sakura, Zoeterwoude, Netherlands) and 5 μM cryo-sections were prepared. These Sections were fixed in 100 % acetone and equilibrated in PBS followed by overnight incubation at 4 °C with anti-VEGF rabbit polyclonal antibody (Santa Cruz Biotechnology Inc., USA) which was diluted with Dako antibody diluents (DAKO, Hamburg, Germany). The cryosections were then incubated with Anti-rabbit Alkaline Phosphatase supervision polymer system (DCS Innovative Diagnostik-Systeme, Hamburg, Germany). Staining was visualized using the Neu Fuchsin substrate Chromogen (DCS Innovative Diagnostik-Systeme, Hamburg, Germany) and were counterstained with Hematoxylin and mounted in Kaisers Glycerol Gelatin (Merck, Darmstadt, Germany). To evaluate the expression of VEGF, all slides were examined and scored by two independent pathologists who were blinded to the animal data. The percentage staining was scored as follows: 0 (No staining, 1 (0-5 %), 2 (6-25 %), 3 (26-50 %), 4 (51-75 %), 5 (76-100 %).

### Statistical analysis

The mean tumor growth ratio (V2/V1, V2 tumor volume after treatment and V1 tumor volume before treatment) by MRI and the mean expression ratio (VEGF/β-actin) level of VEGF by Western blot from each group were measured and the significance of differences between the two groups were analyzed using the paired-*t*-test, the statistical software used was GraphPad Prism (version 3.02, La Jolla, CA, USA). Immunohistochemical staining of VEGF was evaluated using descriptive and semi-quantitative methods. The differences between both groups in the Western-Blot analysis and Immunohistochemical analysis were tested for statistical significance using the unpaired-*t*-test and the Wilcoxon signed rank test respectively. Differences with a p value less than 0.05 were considered statistically significant.

## Results

### MRI examination

**Fig. 1:**
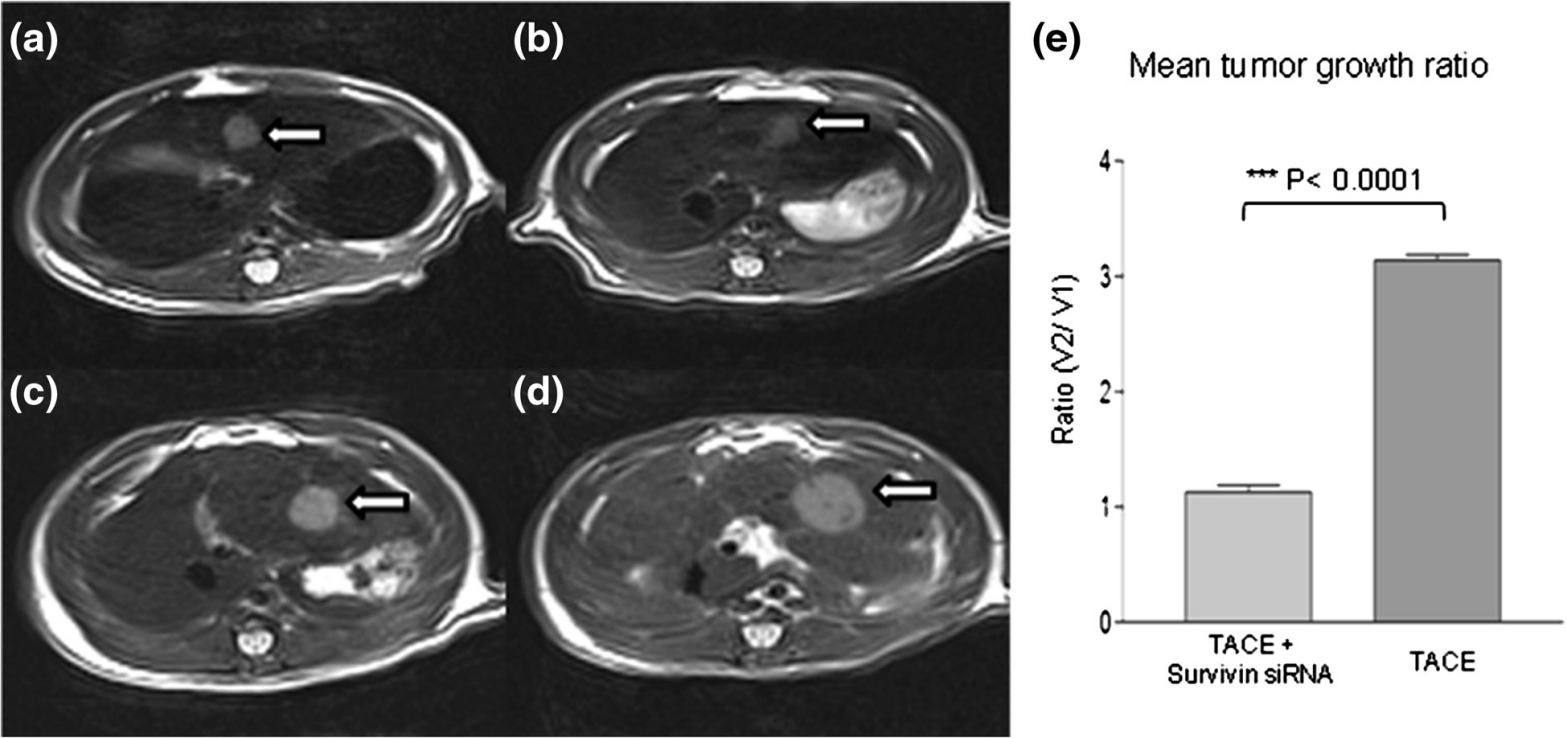
Transverse unenhanced T2-weighted TSE MR images of solid liver tumor in a group A (TACE+ survivin siRNA) (images a and b) and group B (control group, TACE alone) (images c and d) in ACI rat. 3870/80 matrix was acquired. a Pretreatment shows a small hyperintense tumor (arrow) in the left lateral liver lobe (0.55 × 0.54 cm). **b** Posttreatment demonstrates the same hyperintense tumor lesion (arrow) (0.55 × 0.53 cm) and has inhomogeneous hypointense area corresponding to intratumoral necrosis. The growth of hepatic tumor is noticeably inhibited after therapy. **c** Pretreatment shows a small hyperintense tumor (arrow) in the left lateral liver lobe (0.69 × 0.68 cm). **d** Posttreatment shows the same (1.14 × 0.98 cm) tumor (arrow) exhibiting rapid growth compared with its size before therapy. **e** Mean tumor growth ratio of post-treated (V2) and pre-treated tumor (V1) by MRI showed significant difference between group A (TACE + survivin siRNA) vs group B (control group, TACE alone)

Tumor implantation was successful in 100 % of the rats. Most tumors appeared homogeneous and were hypointense on T1-weighted images and hyperintense on T2-weighted images prior to treatment, after treatment the tumors appeared inhomogenous. After different interventional treatments, intrahepatic metastases developed in two of the 10 rats in group B. The means of the volume ratios (V2/V1) were 1.1313 ± 0.1381 in group A, and 3.1911 ± 0.1393 in group B. Compared to group B, group A showed a statistically significant reduction of the tumor growth within the period of observation (*p* < 0.0001) (Fig. 1).

### Western blot analysis

**Fig. 2:**
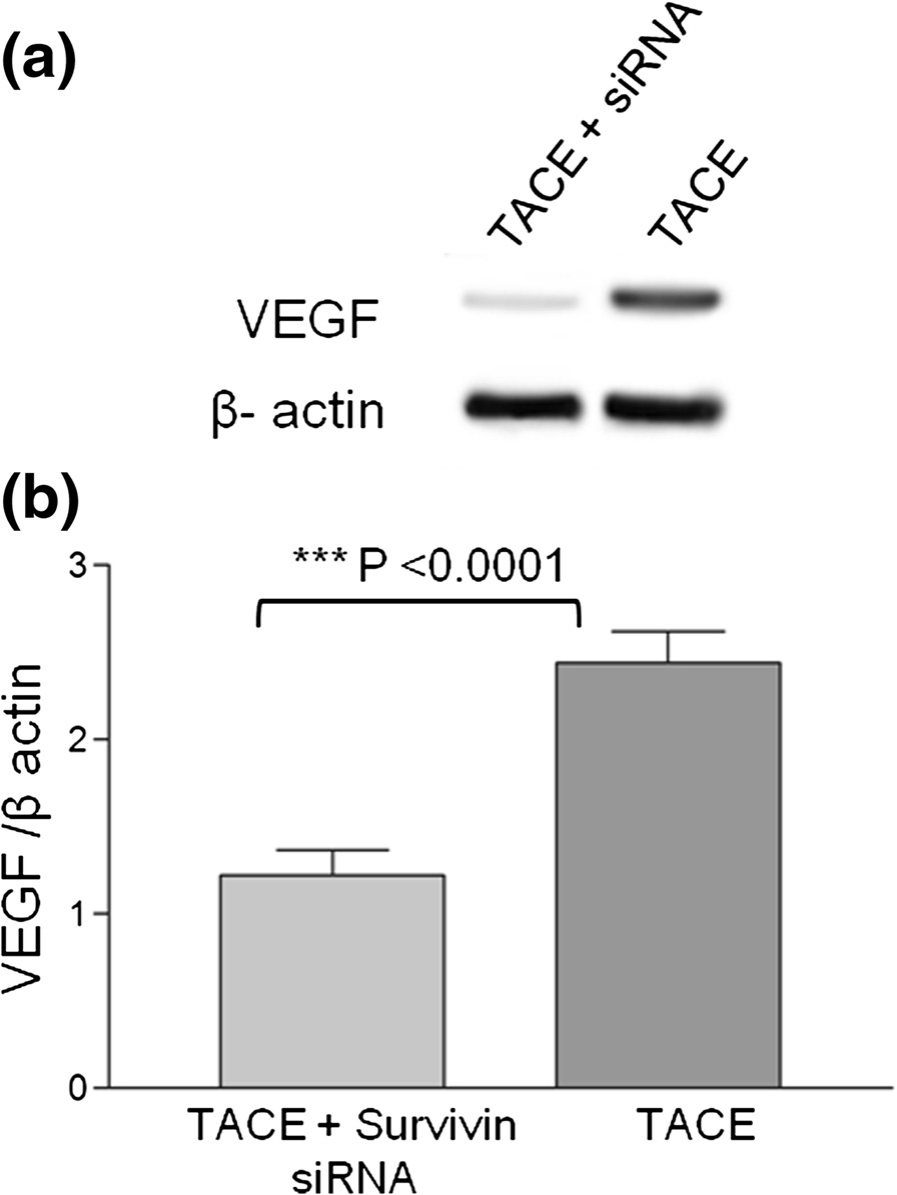
Inhibitory effects of survivin siRNA on the expression of VEGF by Western blot analysis. **a** VEGF bands in group A (TACE + survivin siRNA) was suppressed compared to TACE alone (control). Depicted are representative bands from 10 independent experiments (*N* = 10) **(b)** Shows the semi quantification of proteins bands, expressed as ratio of VEGF **/** β-actin. Group A (TACE + survivin siRNA) was down regulated compared to Group B (TACE alone) (*N* = 10)

VEGF expression level was lower in group A (TACE + Survivin siRNA) than in group B (TACE alone). There was a statistically significant difference between group A and group B regarding the VEGF expression by Western blot analysis (*p* < 0.0001) (Fig. 2).

### Immunohistochemical assay

**Fig. 3:**
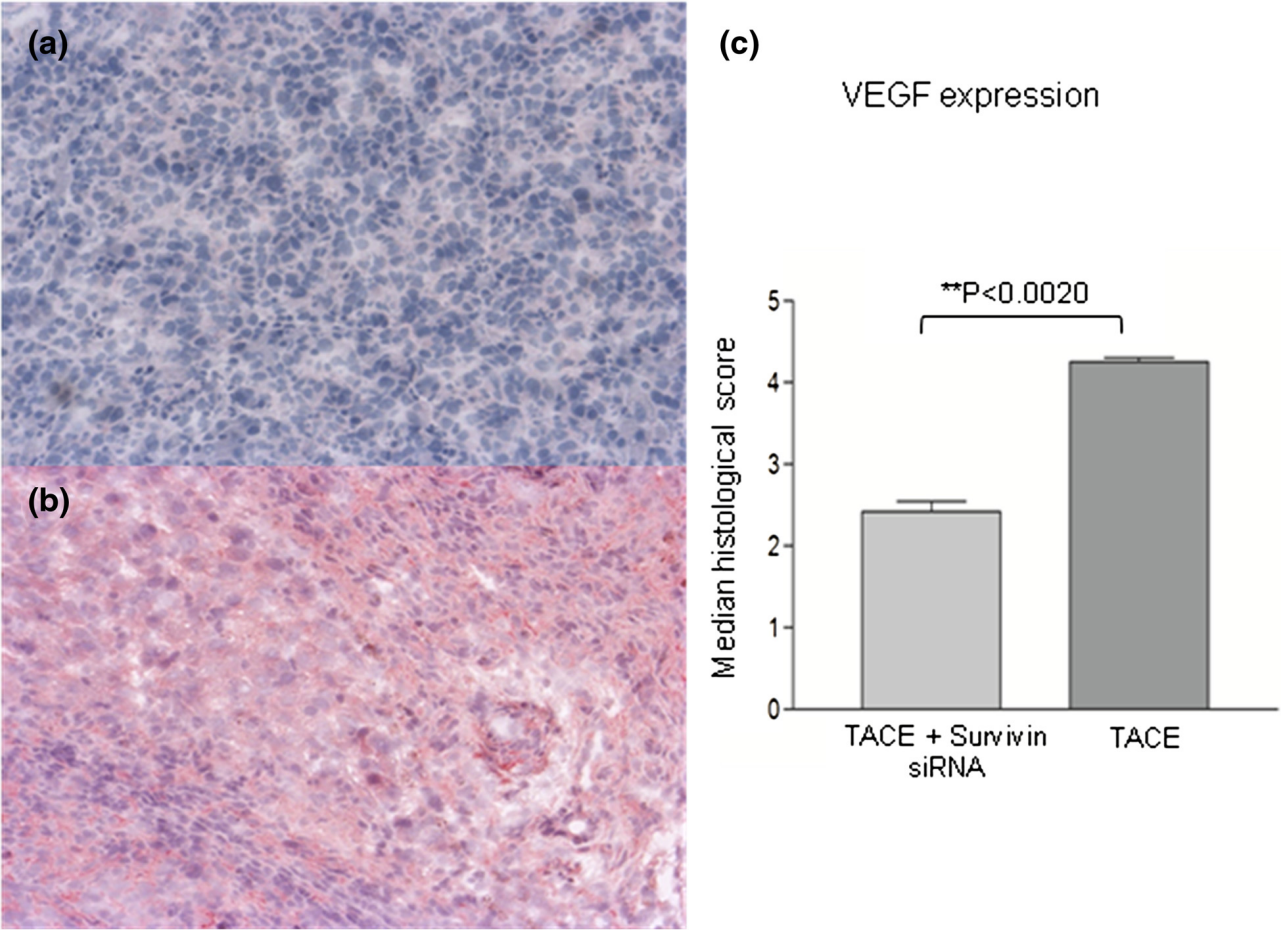
The immunohistochemical staining of VEGF in hepatocellular carcinoma. **a** VEGF staining in hepatocellular carcinoma in the group A (TACE + survivin siRNA) (×100). **b** Significantly higher VEGF staining in hepatocellular carcinoma was observed in group B (control group, TACE alone) than group A (×100). **c** Median histological score of VEGF (*N* = 10)

The angiogenesis of the tumor was evaluated using the Anti-VEGF antibodies. VEGF were expressed in all specimens. The Immuno-expression of the protein was confirmed by the presence of brown stained cytoplasm in tumor cells. Higher expression of VEGF in hepatocellular carcinoma was observed in group B (TACE alone) with a median histological score of 4.250 compared to group A (TACE + Survivin siRNA) with a median histological score of 2.450. The difference between both groups was statistically significant (*p* = 0.0020) (Table 1) (Fig. 3).

**Table 1:** Immunuhistochemical expression of VEGF in hepatocellular carcinoma

Tumor	Tace + Survivin siRNA (*N*=10*)* Median Score	TACE (*N*=10) Median Score	*P* value (Wilcoxon signed rank test)
VEGF	2.450	4.250	0.0020

Scoring: 0 (No staining), 1 (0-5%), 2 (6-25%), 3 (26-50%), 4 (51-75%), 5 (76-100%).

## Discussion

Since the introduction of TACE as a palliative treatment in patients with unresectable HCC, it has become one of the most common forms of interventional therapy [2, 10]. TACE reduces the maximum plasma concentration, lengthens the half-life, and increases the average concentration of chemotherapeutic agents in the tumor [14]. However, post-interventional metastasis and recurrence of tumors have hindered the curative effect of interventional therapeutic procedures and the long-term survival rates [2]. Moreover, a study by Kobayashi et al. [15] showed that the serum concentration of VEGF was markedly increased in patients following embolization.

Biologically, Survivin has been shown to inhibit apoptosis, enhance proliferation and promote angiogenesis. High expression levels of Survivin correlate with an increased rate of tumor recurrence and resistance to chemotherapy [16]. Several in vitro and in vivo studies have indicated that Survivin down-regulation is able to sensitize human tumor cells of different histologic origins to conventional chemotherapeutic drugs [17]. Another important point to notice is that Survivin plays an important role in response to Radiotherapy too; a high level of Survivin has been shown to increase both the resistance to Radiotherapy and the incidence of local recurrence in rectal cancer patients [18] and is associated with worsened survival in patients treated with definitive Radiotherapy for cervical cancer [7, 19]. Similarly, it might be suggested that Survivin expression might have a similar effect on patient response to Radioembolization of hepatic malignancy and that inhibiting Survivin expression using Survivin siRNA might have a favorable effect on patient response to Radioembolization; similar to what was previously reported by Yang et al. [20] where the authors reported an enhanced radiosensitivity in human hepatoma cells both in vitro and in vivo in response to Survivin downregulation by Survivin siRNA. Hence combining Survivin siRNA and Radioembolization might be associated with an improved response to Radioembolization, still the medical literature is lacking such a study.

As Survivin is not a cell surface protein and does not have an intrinsic enzymatic activity, targeting of Survivin for therapeutic purposes might be expected to be difficult. In addition, crystallographic data has revealed few potential drug able sites on Survivin protein [21]. Because of the up-regulation of Survivin in malignancy and its key role in apoptosis, proliferation and angiogenesis, Survivin is currently attracting considerable attention as a new target for anti-cancer therapies. Strategies under investigation to target Survivin include antisense oligonucleotides, small interfering RNA (siRNA), ribozymes, immunotherapy and small molecular weight molecules [22].

Recently, siRNA technology holds a great promise as a therapeutic intervention for targeted gene silencing in cancer. RNA interference (RNAi) is a biological mechanism whereby the presence of double-stranded RNA (dsRNA) interferes with the expression of a particular gene that shares a homologous sequence with the dsRNA. Recent studies have provided insights into the molecular mechanisms of RNAi, in which dsRNA induces the silencing of homologous mRNA. In the cytoplasm of mammalian cells, an enzyme known as Dicer initiates RNA silencing by the breakdown of long dsRNA to generate siRNA of about 21–23 nucleotides in length. The resulting siRNAs are incorporated into an RNA-induced silencing complex (RISC) and unwound into a single-stranded RNA (ssRNA), which is followed by the degradation of ssRNA [23]. siRNA is 10–100-fold more potent for gene silencing, making it attractive tool for silencing of target genes in cancer [24]. An Adenovirus-mediated siRNA expression vector was reported to decrease Survivin expression of the established HCC tumor in nude mice. In vitro study showed that stable Survivin-knockdown inhibited cancer cell proliferation, enhanced apoptotic susceptibility, arrested cell cycle in the G1 phase and resulted in an apparent mitotic catastrophe. An additional in vivo study showed that intra-tumoral injection of Adenovirus-delivered Survivin siRNA suppressed tumor growth by spontaneous apoptosis of cancer cells and significantly prolonged animal survival [25]. However, in vivo systemic delivery of siRNA-based therapeutics to tumor tissues/cells remains a challenge. The major limitations against the use of siRNA as a therapeutic tool are its degradation by serum nucleases, poor cellular uptake, nonspecific immune stimulation and rapid renal clearance following systemic administration [24, 26].

The current study was designed to reduce tumor progression by using a combination of transarterial administration of Survivin siRNA and TACE using the sandwich technique in an animal model of HCC. We did not include intravenous delivery approach because the transarterial route was much more efficient in the treatment of HCC. In the current study however we did not evaluate the expression of Survivin directly in the Hepatoma 3924a in addition we did not assess the degree of suppression of Survivin expression in response to treatment for several reasons. First the expression of Survivin has been reported to occur in several HCC cell lines and in human HCC tissue [27], it has been reported to be highly expressed in the vast majority of human cancers including HCC [28]. Second we relied on the radiologic oncologic assessment of the therapeutic effect of Survivin siRNA rather than on its direct assessment. A direct assessment of the suppression of Survivin was rather outside the scope of the current study. Finally Survivin expression has been shown to significantly correlate with VEGF expression in HCC [29], hence we relied on this correlation to indirectly assess Survivin suppression (through VEGF assessment). Still we regard this as one of the limitations of the study and recommend the subject for further evaluation in future studies assessing not only the response based on radiologic evidence but based on direct assessment of Survivin. Another limitation of the current study is the lack of a negative control group (receiving si-control RNA).

Survivin has been shown to play an important role in the regulation of expression of VEGF in breast cancer lymphatic metastases [30]. In addition it has been shown to significantly correlate with the expression with VEGF in hepatocellular carcinoma and promote the expression of VEGF [28, 29]. Our experimental results demonstrated that groups A (TACE + Survivin siRNA) showed a significant reduction of tumor growth in the period of observation compared to the control group (TACE alone). Higher Immunohistochemical expression of VEGF in hepatocellular carcinoma was observed in group B (TACE alone) than that of group A (TACE + Survivin siRNA) (*P* < 0.01). The invasive progression of tumor cells in group A was noticeably inhibited compared with group B. This complex targeting treatment method seemed to overcome the insufficiency of TACE and improve the overall therapeutic effects.

## Conclusion

In conclusion, the additional injection of Survivin siRNA to the routine protocol of TACE has shown to be safe and effective in an animal model of HCC in rats. This combined therapeutic therapy noticeably inhibited the growth of the hepatic carcinoma in rats compared with TACE alone. Further experimental studies will be required to fully understand the benefits and risks of this strategy for treating HCC before transferring it to human studies.

### Ethics approval and consent to participate

The current animal study was approved by the Regional Administrative Authority in Darmstadt, Germany.

### Consent for publication

The current study does not contain any personal materials that require patient approval.

### Availability of data and materials

Due to organizational restrictions the data will not be available.

## Abbreviations

HCC: hepatocellular carcinoma
SDS-PAGE: sodium dodecyl sulfate polyacrylamide gel electrophoresis
siRNA: small interfering RNA
TACE: transarterial chemoembolization
VEGF: vascular endothelial growth factor expression
